# Genetic association study of mitochondrial polymorphisms in neovascular age-related macular degeneration

**Published:** 2013-05-29

**Authors:** Julien Tilleul, Florence Richard, Nathalie Puche, Jennyfer Zerbib, Nicolas Leveziel, Jose Alain Sahel, Salomon Yves Cohen, Jean-Francois Korobelnik, Josue Feingold, Arnold Munnich, Josseline Kaplan, Jean-Michel Rozet, Eric H. Souied

**Affiliations:** 1 Department of Ophthalmology, Hôpital Intercommunal de Creteil, University of Paris Est Creteil, France; 2 Department of Genetics, INSERM U781, Hôpital Necker Enfants Malades, University of Paris Descartes, Paris, France; 3 University Lille Nord of France, INSERM, UMR744, Institut Pasteur of Lille, Lille, France; 4 Department of Ophthalmology, Institut de la Vision, Inserm, Pierre et Marie Curie University, Paris, France; 5 Ophthalmologic Centre of Imaging and Laser, Paris, France; 6 Ophthalmology, Hôpital Pellegrin CHU de Bordeaux, Université Bordeaux 2, INSERM U897, Bordeaux, France

## Abstract

**Purpose:**

Age-related macular degeneration (AMD) is a multifactorial disease involving genetic and environmental factors. Most of the genetic factors identified so far involve the nuclear genome. Recently, two studies in North America and Australia reported an association between advanced AMD and the mitochondrial T2 haplogroup. Our purpose was to assess this association in a large French population.

**Methods:**

This case control study included 1,224 patients with neovascular AMD and 559 controls with normal fundus. Mitochondrial DNA polymorphisms at and around nucleotides 4917, 11,812, and 14,233 were determined using PCR amplification and direct sequencing of mitochondrial DNA.

**Results:**

No association was found between the mitochondrial T2 haplogroup and neovascular AMD in the French population: 94/1,152 patients with neovascular AMD had the T2 haplogroup (8.2%) versus 34/482 controls (7.1%; odds ratio=0.9 [0.5–1.5], p=0.66).

**Conclusions:**

An association between AMD and the T2 haplogroup, previously described in North American and Australian populations, was not confirmed in a large French population.

## Introduction

Age-related macular degeneration (AMD) is the leading cause of blindness in Western countries. AMD is characterized by a progressive loss of central vision attributable to degenerative and/or neovascular changes that occur in the interface between the neural retina and the underlying choroid. Late stage AMD includes two main subtypes with unequal frequencies: atrophic AMD accounts for one third of cases whereas two thirds of patients suffer from neovascular AMD [1]. Atrophic AMD is characterized by a gradual degeneration of the macular retinal pigment epithelial layer that leads to a slowly progressive loss of macular photoreceptors and a gradual loss of central vision [2]. Neovascular or exudative AMD results from choroidal neovascularization through Bruch's membrane and rapidly alters central vision, though progressive damage to photoreceptor cells is caused by blood and protein leakage [3-5].

AMD is a multifactorial disease involving genetic and environmental risk factors. Over the last few years, efforts to identify the genetic factors related to AMD have resulted in the identification in the nuclear genome of high-risk alleles in complement factor H (*CFH*) [6-8] and *ARMS2* [9,10], and of susceptibility alleles in apolipoprotein E (*ApoE*), pigment epithelium-derived factor (*PEDF*), clusterin, Scavenger receptor class B member 1 (*SRB1*), hepatic lipase (*LIPC*), tissue inhibitor of metalloproteinases-3 (*TIMP3*), and *VEGF* [11-22]. Recently, in the United States, risk alleles were found in mitochondrial DNA with an increased frequency of the mitochondrial haplogroup T in patients with AMD [23]. Subsequently, it was shown that, in mixed populations from the United States and Australia, the association with advanced AMD (neovascular and atrophic) was driven by variants of respiratory Complex I that uniquely characterize haplogroup T2: 4917G (non-synonymous), 11812G (synonymous), and 14233G (synonymous) [24]. These findings gave further support to a correlation between levels of respiratory chain bioproducts of reactive oxygen species (ROS), oxidative stress, and degenerative changes typical of AMD.

The aim of the present study was to assess whether haplogroup T2 variants conferred a high risk for neovascular AMD in a French population. Our study was retrospective (patients/DNA samples were previously recruited). We decided to focus on patients with neovascular AMD to screen a large homogeneous population.

## Methods

### Patients

This case-control study included 1,224 Caucasian patients with AMD with choroidal neovascularization (mean age ± standard deviation [SD] at AMD diagnosis was 78.8±7.4 years) who were recruited between 2005 and 2009 at four French ophthalmic centers with the potential to recruit a high number of patients with AMD: the Ophthalmology Eye Clinic of Creteil, Pellegrin Hospital in Bordeaux, Quinze-Vingts Hospital, and the Centre of Imaging and Laser of Paris. Inclusion criteria were i) women or men aged 55 or older and ii) exudative AMD in at least one eye. The exclusion criterion was the presence of other retinal disease (e.g., diabetic retinopathy, high myopia, or macular dystrophies). Patients underwent a complete ophthalmologic examination including i) best-corrected visual acuity measurement, ii) fundus examination with retinal photographs, iii) fluorescein angiography (Topcon 50IA camera, Tokyo, Japan) and, if needed, indocyanine green angiography (HRA, Heidelberg, Germany), and iv) optical coherence tomography (OCT, Carl Zeiss Meditec, Inc., Jena, Germany). A questionnaire about medical history and smoking was completed. Written informed consent was obtained, as required by French bioethical legislation and the local ethics committee (*CCPPRB Henri Mondor*), in agreement with the Declaration of Helsinki for research involving human subjects. The study and data collection methods were approved by our Institutional Review Board (*CCPPRB Henri Mondor*).

### Controls

The study population included 559 Caucasian controls (mean age±SD was 67.7±7.8 years) recruited consecutively at the Eye Ophthalmology Clinic of Creteil between 2002 and 2010. Fundus examination and retinal photography were performed for each control to exclude any abnormal feature on the macula (no drusen, no pigment epithelium alteration, no hemorrhage, no exudate, no macular edema, no myopic maculopathy). All retinal photos were graded by senior ophthalmologists (ES, SYC, JAS, JFK). All controls were recruited among patients who had had cataract surgery. Information about their medical history, including smoking, was obtained.

### Genotyping

Genomic DNA was extracted from 10 ml blood leukocytes using the Illustra kit, according to the manufacturer’s protocol (GE Healthcare, Little Chalfont Buckinghamshire, UK). Methods for genotyping *CFH* and *ARMS2* have already been described [25,26]. Mitochondrial DNA was genotyped using primers designed to amplify the genes encoding the NADH dehydrogenase subunits 2 *(ND2*), 4 (*ND4*), and 6 (*ND6*), respectively (Table 1). Purified PCR fragments were directly sequenced, using the BigDye Terminator Cycle Sequencing Kit v3.1 (Applied Biosystems, Foster City, CA). The *ND4* G11778A mutation and the single nucleotide polymorphisms (SNPs) A11812G, A11914G, G12007A and T14167C, T14110C, T14180C, T14182C, T14212C, G14364A, and T14470C/A in *ND4* and *ND6*, respectively, were analyzed.

**Table 1:** Primers used to amplify mitochondrial DNA

Gene	Polymorphisms analyzed	Forward primer	Reverse primer
ND2	A4917G	5′ CCTGCTTCTTCTCACATGAC 3′	5′ GGGTCTGGTTTAATCCACCT 3′
ND4	A11812G, A11914G, G12007A	5′ CTTACATCCTCATTACTATTC 3′	5′ AAACTATATTTACAAGAGGAAAAC 3′
ND6	A14233G, T14110C, T14167C, T14180C, T14182C, T14212C, G14364A, T14470C/A	5′ ACCTGCCCCTACTCCTCCTA 3′	5′ GTAGTTGAAATACAACGATGG 3′

### Statistical analysis

The Hardy–Weinberg assumption was assessed with the standard method comparing the observed number of subjects in the different genotype categories with the expected number under the Hardy–Weinberg equilibrium for the estimated allele frequency, and testing with a Pearson goodness-of-fit statistic with the χ^2^ with 1 degree of freedom. The χ^2^ test was used to compare categorical allelic and genotype distributions between cases and controls. Logistic regression was used to estimate the adjusted odds ratio (OR) with a 95% confidence interval (95% CI). Models were adjusted for age, sex, tobacco status, and *CFH* and *ARMS2* genotypes. P values for mitochondrial polymorphisms were presented without and with Bonferroni correction for 11 SNPs. As all patients were bearers of the G allele, no statistical analysis was performed, and we did not consider the G11778A polymorphism.

## Results

The genotypes of the 1,224 exudative AMD cases and 559 controls for the rs1061170 (Y402H, *CFH*), rs10490924 (*ARMS2*), mitochondrial 4917 (mt4917; *ND2*), mt11812 (*ND4*), and mt14470 (*ND6*) SNPs, respectively, are shown in Table 2. The genotypic distributions of the *CFH* and *ARMS2* SNPs were significantly different between the cases and controls (p<0.0001). The Y402H *CFH* and *ARMS2* rs10490924 polymorphisms were in Hardy–Weinberg equilibrium in the control group. The genotypes of the cases and controls for mitochondrial polymorphisms are shown in Table 3. No association was found between the mitochondrial 4917G and 11812G polymorphisms (defining mitochondrial haplogroup T2) and neovascular AMD before or after adjustment for age, sex, smoking status, and *CFH* and *ARMS2* (OR=0.9 [0.5–1.6]; Table 3 and Table 4). Regarding mitochondrial polymorphisms, A11914G, G12007A, T14110C, T14180C, T14182C, T14212C, A14233G, G14364A, and the G11778A mutation lying in the amplified regions, we found no significant association with AMD (Table 5).

**Table 2:** Demographic characteristics of the population

n	Controls	Cases	p
	559	1224	
Men, n (%)	216 (38.6%)	414 (33.8%)	0.05
Age, years, m (sd)	67.7 (7.8)	78.8 (7.4)	<0.0001
Tobacco, n (%)	236 (42.2%)	461 (37.7%)	0.07

**Table 3:** Genetic characteristics of the population.

Polymorphisms	Controls	Cases	p	corrected p†
n	559	1224		
**CFH**				
TT, n(%)	209 (37.7%)	266 (21.8%)	<0.0001	
CT	268 (48.3%)	612 (50.1%)		
CC	78 (14.0%)	344 (28.1%)		
**ARMS2**				
GG	330 (60.2%)	373 (30.6%)	<0.0001	
GT	192 (35.1%)	572 (46.9%)		
TT	26 (4.7%)	274 (22.5%)		
**4917**				
A	509 (91.5%)	1103 (90.1%)	0.34	1
G	47 (8.5%)	121 (9.9%)		
**11812**				
A	450 (92.8%)	1058 (91.8%)	0.52	1
G	35 (7.2%)	94 (8.2%)		
T2 (4917G11812G)	34 (7.1%)	94 (8.2%)	0.45	1
“Not T2” (4917A11812A or 4917G11812A)	448 (92.9%)	1058 (91.8%)		
**14470**				
T	528 (95.2%)	1129 (97.7%)	0.02*	0.22
C	18 (3.2%)	19 (1.6%)		
A	9 (1.6%)	8 (0.7%)		
C or A	27 (4.9%)	27 (2.3%)	0.006	0.07
**G11778A**				
G	484 (100%)	1139 (100%)		
**A11914G**				
G	478 (97.0%)	1129 (98.2%)	0.12	1
A	15 (3.0%)	21 (1.8%)		
**G12007A**				
G	487 (98.4%)	1128 (99.1%)	0.19	1
A	8 (1.6%)	10 (0.9%)		
**T14167C**				
C	507 (90.7%)	1124 (98.8%)	0.13	1
T	52 (9.3%)	87 (7.2%)		
**T14110C**				
T	556 (99.6%)	1200 (99.9%)	0.24*	1.00*
C	2 (0.4%)	1 (0.1%)		
**T14180C**				
C	558 (99.2%)	1199 (99.4%)	0.45*	1.00*
T	1 (0.2%)	7 (0.6%)		
**T14182C**				
T	542 (97.0%)	1165 (96.4%)	0.58	1
C	17 (3.0%)	43 (3.6%)		
**T14212C**				
T	556 (99.5%)	1206 (99.5%)	1.00*	1.00*
C	3 (0.5%)	6 (0.5%)		
**G14364A**				
G	553 (99.1%)	1174 (99.2%)	1.00*	1.00*
A	5 (0.9%)	10 (0.8%)		

* exact test of Fisher † p adjusted with Bonferroni correction

**Table 4:** Crude and adjusted Odds Ratios of having neovascular AMD in mitochondrial T2 haplogroup and according to mt14470 polymorphism

4917G and 11812G	Crude OR [95% CI]	p	Model adjusted for age, sex, tobacco CFH and ARMS2 OR [95% CI]	p
4917G	1.2 [0.8–1.7]	0.34	1.0 [0.6–1.5]	0.88
11812G	1.1 [0.8–1.7]	0.52	1.0 [0.3–3.0]	0.99
4917G 11812G (=T2 haplogroup)	1.2 [0.8–1.8]	0.45	0.9 [0.5–1.6]	0.74
14470	Crude OR [95% CI]	p	Model adjusted for age, sex, tobacco OR [95% CI]	p
14470T	1 (ref)	0.023	1 (ref)	0.36
14470C	0.5 [0.3–0.9]		0.6 [0.2–1.3]	
14470A	0.4 [0.2–1.1]		0.7[0.2–2.4]	
14470C or A	0.5 [0.3–0.8]	0.006	0.6 [0.3–1.2]	0.23

**Table 5:** Crude and Adjusted Odds Ratios of having neovascular AMD according to different mitochondrial polymorphisms

Polymorphisms	Crude OR [95% CI]	p	Model adjusted for age, gender, tobacco CFH and ARMS2 OR [95% CI]	p
A11914G	0.6 [0.3–1.2]	0.13	0.40 [0.2–1.1]	0.07
G12007A	0.5 [0.2–1.4]	0.19	0.3 [0.1–0.9]	0.04
T14167C	0.8 [0.5–1.1]	0.13	0.7 [0.4–1.1]	0.09
T14110C	0.2 [0.1–2.6]	0.23	0.8 [0.1–10.1]	0.85
T14180C	0.3 [0.1–2.5]	0.27	0.3 [0.1–5.3]	0.4
T14182C	1.2 [0.7–2.1]	0.58	1.1 [0.5–2.3]	0.82
T14212C	0.9 [0.2–3.7]	0.91	1.4 [0.3–7.7]	0.69
G14364A	0.9 [0.3–2.8]	0.91	0.9 [0.2–3.6]	0.91

Conversely, regarding the T14470C/A polymorphism, we found higher frequencies of 14470C or 14470A alleles in controls versus cases (crude OR=0.5 [0.3–0.8], p=0.006). However, the results were not statistically significant after Bonferroni correction (p=0.07) and when the model was adjusted for age, sex, smoking status, and *CFH* and *ARMS2* (OR=0.6 [0.3–1.2], p=0.23, Table 4). In addition, we found more people with 14167C polymorphisms in the control group than in the AMD patient group, but the difference was not significant when adjusted for age, sex, smoking status, and *CFH* and *ARMS2* status (OR=0.7 [0.4–1.1], p=0.09 for C *versus* T allele bearers).

## Discussion

AMD is a multifactorial disease that involves environmental and genetic factors. Over the last decade, the identification of major risk alleles for AMD suggested that inflammation and lipid homeostasis were the main pathways involved in the disease. Recently, evidence linking allelic variation in mitochondrial DNA with AMD was found, supporting the long-suspected notion of oxidative stress contributing to the pathogenesis of the disease. The link between oxidative stress, mitochondrial metabolism, and the aging process is well established [27-29]. Reactive oxygen species (ROS) are mainly produced by the respiratory chain in the mitochondria where, at high concentrations, they may injure the mitochondrial genome and thus increase the age-related disease sequelae. The high frequency of some mitochondrial haplogroups in centenarians in Europe (J and U haplogroups) and Asia (D4a) [30-33] and the potential association between U or H haplogroups and Alzheimer disease [34,35] supports this hypothesis.

Regarding AMD, the photoreceptor/retinal pigment epithelium (RPE) complex is located in a unique high-oxidative-stress microenvironment due to the generation of high concentrations of light-induced ROS. A body of evidence suggests a major role of oxidative stress response and of mitochondrial dysfunction in the pathogenesis of the disease, including i) RPE lesions in superoxide dismutase 2 (*sod2*) knockdown mice similar to those observed in atrophic AMD [36], ii) decreased mitochondrial respiration with alterations of mitochondrial DNA in the macular RPE of patients with AMD [37], iii) quantitative and qualitative alterations of mitochondrial cristae (ultrastructure) in the RPE cells of 75-year-old patients with age-related maculopathy (ARM), identical to those of 85-year-old ARM-free donor individuals [38]. In 2007, the first case-control association study of mitochondrial variants in age-related maculopathy supported the idea of a decreased risk of ARM in individuals carrying the H haplogroup and an increased risk of soft drusen and RPE abnormalities in those with the J and U haplogroups [39]. Another case control study identified haplogroup J as a risk factor for advanced AMD and haplogroup H as a protective factor [40]. More recently, a whole-mitochondrial genome case-control association study found an increased risk of advanced AMD in carriers of the T2 haplogroup (OR=2.54, p≤0.004) [24]. A decrease in respiratory chain complex I activity has been found in the sperm of individuals with the T haplogroup (defined by the mt4917G polymorphism) [41]. Furthermore, an increased susceptibility to oxidative stress has been related to deficient complex I activity [42]. It is therefore possible that the retinas of individuals with the T haplogroup may be more susceptible to ROS species. In our study, we have not confirmed the association between several mitochondrial polymorphisms and neovascular age-macular degeneration in a large cohort of patients with AMD in France. This discrepancy between previous studies and our work may be explained in part by differences in cohort structures. Indeed, in Canter’s paper, which included age-related maculopathy and advanced AMD, the frequency of the T haplogroup in clinical cases was 15.4% versus 9.9% in our study, while the frequency in the control group was comparable with our results [23]. In SanGiovanni’s paper, the frequency of the mitochondrial T2 haplogroup was much lower in the Age-Related Eye Disease Study (AREDS) control population: 1.8% versus 7.1% in our control population [24]. Indeed, here, we focused on neovascular AMD whereas earlier reports considered heterogeneous patient subgroups in the AREDS and Blue Mountain Eye Study cohorts, respectively, containing neovascular and atrophic AMD. In SanGiovanni’s paper, 35% of the AREDS population had atrophic AMD, with a slightly higher proportion of T2 in this subgroup: 10.8% versus 9.5% in the neovascular AMD subgroup. It is also likely that interactions between genetic and environmental factors that contribute to AMD may differ in American and European populations [43-45]. Regarding oxidative stress, ROS production is influenced by nutrition [46,47]. Owing to the marked differences in dietary habits in the United States and France, it is conceivable that individuals with the same mitochondrial genotypes living in the United States or France may have different susceptibilities to oxidative stress.

However, the lack of association between the T2 haplogroup and neovascular AMD in the French population does not rule out the possible contribution of mitochondrial variants in this population. Other haplogroups such as H12 or U5a have been associated with a decreased stability of complex I [48] and could be associated with AMD in the French population. From this point of view, our results suggest that two mitochondrial variants in *ND6*, 14470A (haplogroup H10) and 14470C (haplogroups B5b1, D4e1a, L1c6, M8a, P1b, U6, and X), may confer protection against neovascular AMD. To confirm this hypothesis, it is necessary to increase the size of the population. Indeed, although the crude odds ratio suggested a protective role of the 14470A and 14470C alleles, the adjusted odds ratio was not significant. This might be correlated with the fact that patients carrying these alleles were younger than those carrying the 14470T allele. Our results should be interpreted with caution. We did not have sufficient power for some polymorphisms of interest in particular for the A11914G, G12007A, T14110C, T14180C, T14182C, T14212C, 14,470, and G14364A polymorphisms. However, for the 4917, 11,812, and T14167C polymorphisms, we had a power of at least 80% to detect an odds ratio of 2 after Bonferroni correction.

In conclusion, no association between the mitochondrial T2 haplogroup and neovascular AMD in French patients was observed, but further studies are required to assess the role of mitochondrial polymorphisms in this disease. The advent of high-throughput sequencing should be of major help in this task by allowing fast sequencing of the entire 16.5 kb mitochondrial genome in a large population of patients with clinically defined AMD.
